# Tannic Acid/Fe(III)-Coated Curcumin Self-Assembled Nanoparticles for Combination Therapy to Treat Triple-Negative Breast Cancer

**DOI:** 10.3390/pharmaceutics17101257

**Published:** 2025-09-25

**Authors:** Jialing Li, Ning Han, Mingyue Ruan, Hongmei Wei, Yunan Dong, Haitong Zhang, Zishuo Guo, Shouying Du, Pengyue Li

**Affiliations:** School of Chinese Materia Medica, Beijing University of Chinese Medicine, Beijing 102488, China; lijialingalg@163.com (J.L.); hanning1989@163.com (N.H.); rmy15207224220@163.com (M.R.); 20240935123@bucm.edu.cn (H.W.); dyyzmn@163.com (Y.D.); 20230935108@bucm.edu.cn (H.Z.); cfplxzks@163.com (Z.G.)

**Keywords:** curcumin, MPNs, self-assembly, photothermal therapy, ferroptosis

## Abstract

**Background/Objectives:** Triple-negative breast cancer (TNBC) exhibits pronounced biological heterogeneity, aggressive behavior, and a high risk of recurrence and metastasis. The conventional treatments for TNBC have notable limitations: surgical resection may leave residual tumor cells; chemotherapy (CT) frequently induces systemic toxicity and drug resistance; and radiotherapy damages surrounding organs and compromises the patients’ immune function. **Methods**: Herein, we designed a carrier-free nanodrug delivery system composed of self-assembled Curcumin nanoparticles (NPs) coated with a tannic acid (TA)/Fe(III) network (denoted as CUR@TA-Fe(III) NPs). We systematically evaluated the in vitro cytotoxicity and photothermal–ferroptosis synergistic therapeutic efficacy of CUR@TA-Fe(III) NPs in 4T1 breast cancer cells, as well as the in vivo antitumor activity using 4T1 tumor-bearing mouse models. **Results:** CUR@TA-Fe(III) NPs had high drug loading efficiency (LE) of 27.99%, good dispersion stability, and photothermal properties. Curcumin could inhibit the growth of 4T1 cancer cells, while TA-Fe(III) efficiently converted light energy into heat upon exposure to near-infrared (NIR) light, leading to direct thermal ablation of 4T1 cells. Additionally, TA-Fe(III) could supply Fe(II) via TA, increase intracellular Fe(II) content, and generate reactive oxygen species (ROS) through the Fenton reaction, in turn inducing lipid peroxidation (LPO), a decrease in mitochondrial membrane potential (MMP), and glutathione depletion, eventually triggering ferroptosis. **Conclusions:** This treatment strategy, which integrates CT, PTT, and ferroptosis, is expected to overcome the limitations of traditional single-treatment methods and provide a more effective method for the treatment of TNBC.

## 1. Introduction

Triple-negative breast cancer (TNBC) lacks the expression of estrogen receptor, progesterone receptor, and human epidermal growth factor receptor 2, rendering it unresponsive to endocrine and anti-HER2 targeted therapies. TNBC exhibits pronounced biological heterogeneity and aggressive behavior, increasing the risk of early recurrence and metastasis in patients [1]. Current treatments for early-stage TNBC have notable limitations: surgical resection may leave residual tumor cells, chemotherapy (CT) frequently induces systemic toxicity and drug resistance, and radiotherapy damages surrounding organs and compromises the patient’s immune function. Therefore, there is a need to find a therapy that can achieve complete tumor ablation while minimizing damage to adjacent normal tissues.

Curcumin, a natural compound extracted from the plant Curcuma longa, is abundant in the Indian subcontinent and Southeast Asia [2] and exerts antioxidant effects on normal tissues by scavenging excessive ROS, suppressing oxidative stress, and protecting cells against oxidative injury [3,4]. Experimental studies have demonstrated the efficacy of Curcumin in eliminating various malignancies [5,6,7]. Specifically, Curcumin has been shown to increase ROS levels and induce ferroptosis in colon cancer, lung cancer, and estrogen receptor-positive breast cancer [8,9,10,11]. The opposite effects of Curcumin on tumor and normal cells, along with its ability to induce ferroptosis, suggest that it may represent a class of therapeutic agents distinct from traditional chemotherapeutics. Nevertheless, the ability of Curcumin to induce ferroptosis in TNBC remains undetermined. Additionally, Curcumin has low water solubility and poor chemical stability [2]. A variety of carrier-based nanosystems have been developed for Curcumin delivery, but they usually suffer from low drug loading efficiency (LE) [12]. Recently, self-assembled nanoparticles (NPs) of drugs have been prepared via the solvent-antisolvent method to achieve high LE [13,14]. However, experiments have shown that the zeta potential of self-assembled Curcumin nanoparticles is lower, making them unable to disperse stably and thus requiring modification or stabilization.

Metal–phenolic networks represent a class of supramolecular network architectures that function through the coordination of polyphenols and metal ions. Research on such networks can be traced back to the pioneering work by Caruso and his team [15], who discovered that tannic acid (TA) could complex with Fe(III) through one-step assembly on various substrates to prepare various films and particles. The coating method is simple and rapid and requires no special equipment. So the method will not cause degradation of the substrate, especially drugs. TA and Fe(III) are also readily available and inexpensive. For the chemical structure of TA, it has a central glucose molecule at its core, with all of its connected hydroxyl groups linked to multiple gallic acid molecule units through hydroxyl ester group bonds. Three galloyl groups from TA can react with each Fe(III) to form a stable complex [16], allowing each TA molecule to react with several Fe(III) centers to be coated on substrates without a redundant embellishment process. Furthermore, TA-Fe(III) is a promising photothermal agent which can generate heat upon near-infrared (NIR) light irradiation, with comparable photothermal stability to other photosensitizers and the capacity to precisely ablate neoplastic tissues [17,18]. Additionally, the TA-Fe(III) network not only demonstrates favorable photothermal characteristics, but also exhibits pH-responsive release behavior. This pH-dependent property facilitates the controlled release of encapsulated bioactive agents [19,20,21]. TA can provide an acidic environment that facilitates the conversion of Fe(III) to Fe(II), thereby enhancing the rate of the Fenton reaction in the tumor microenvironment. This means TA-Fe(III) could function as an endogenous reservoir for ferric and ferrous ions, and could therefore be capable of inducing ferroptosis in a concentration-dependent manner [22,23]. Ferroptosis, a form of cell death, is characterized by excessive generation of reactive oxygen species (ROS) via the Fenton reaction, depletion of glutathione (GSH), mitochondrial damage, and the accumulation of lipid peroxidation (LPO), among which the accumulation of LPO can be considered a typical feature [24,25].

**Scheme 1:**
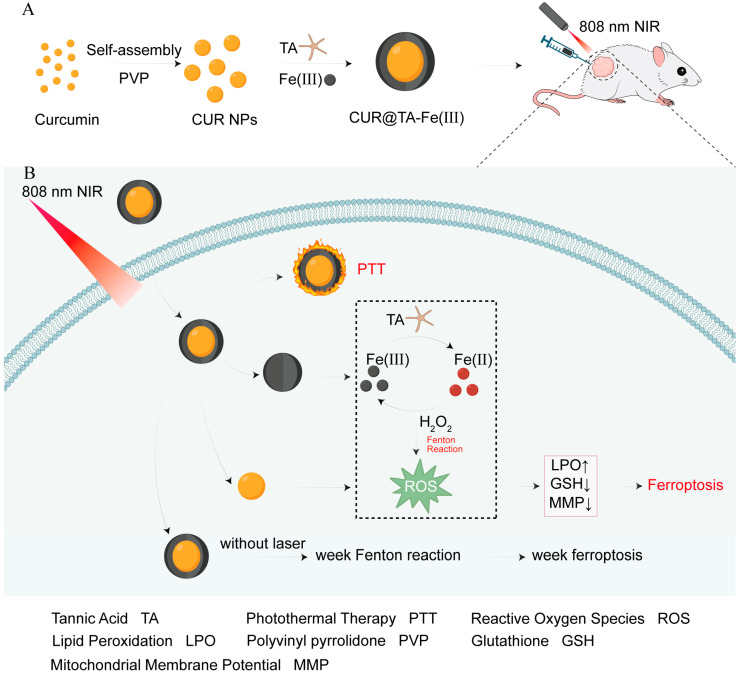
(**A**) Procedure for preparation of CUR@TA-Fe(III) NPs. (**B**) Schematic illustration of the nanosystem for combining antitumor chemotherapy, photothermal therapy, and ferroptosis.

In this work, we developed a simple method to prepare TA-Fe(III) network-coated Curcumin nanoparticles, named CUR@TA-Fe(III) NPs as showed in Scheme 1. This nanosystem exhibited high LE and good dispersing stability and was more easily taken up by 4T1 cells. Curcumin inhibited the growth of 4T1 tumor cells and increased the levels of ROS. Under 808 nm laser irradiation, the TA-Fe(III) coating converted light energy into heat and ablated the 4T1 cells. Additionally, the TA-Fe(III) network could continuously provide ferric and ferrous ions to induce ferroptosis. First, TA reduced Fe(III) to Fe(II), and then, the hydrogen peroxide (H_2_O_2_) in the tumor microenvironment oxidized Fe(II) back to Fe(III) along with ROS production, which is called the Fenton reaction. Furthermore, the heat generated by photothermal therapy (PTT) enhanced the reaction cycle and facilitated a continuous Fenton reaction, thus significantly increasing the levels of ROS to induce ferroptosis. It can be expected that CUR@TA-Fe(III) NPs will effectively integrate PTT with enhanced ferroptosis mechanisms to eliminate tumor cells. This study provides a promising strategy for the combination of Curcumin-mediated chemotherapy with TA-Fe(III)-based photothermal therapy and ferroptosis, aimed at improving therapeutic efficacy.

## 2. Materials and Methods

### 2.1. Materials

Curcumin (AR, R015675) was purchased from Luoen Biotechnology Co., Ltd. (Shanghai, China). Dimethyl sulfoxide (DMSO) was purchased from Modern Oriental Technology Development Co., Ltd. (Beijing, China). Tannic acid (AR, S30456) was purchased from Yuanye Biotechnology Co., Ltd. (Shanghai, China). FeCl_3_ (AR, I811935) was purchased from Macklin Biochemical Technology Co., Ltd. (Shanghai, China). Polyvinylpyrrolidone (PVP, Type K-30, molecular weight at 40,000) was purchased from Yuanye Biotechnology Co., Ltd. (Shanghai, China). Polyvinyl alcohol (average degree of Polymerization at 1700, degree of alcoholysis at 87.0–89.0 mol/mol) was purchased from Macklin Biochemical Co., Ltd. (Shanghai, China). Hydroxypropyl methylcellulose (HPMC, viscosity at 400 mPa·s) was purchased from Aladdin Industrial Corporation. (Shanghai, China). Pluronic F-68 (F68) and Pluronic F-127 (F127) were purchased from Yuanye Biotechnology Co., Ltd. (Shanghai, China). Purified water was purchased from Wahaha Company (Hangzhou, China). Acetonitrile (chromatographic grade) was purchased from Thermo Fisher (Waltham, MA, USA). The Calcein/PI Cell Viability/Cytotoxicity assay kit, DCFH-DA probe, C11-Bodipy 581/591 probe and GSH kit were purchased from Beyotime Biotech Co., Ltd. (Shanghai, China). The FerroOrange probe and JC-1 probe were purchased from Beijing Solarbio Science & Technology Co., Ltd. (Beijing, China).

Experimental animals: SPF balb/c mice (female, 8 weeks, weighing 18 g) were provided by Beijing Sibeifu Laboratory Animal Technology Co., Ltd., license number: SCXK (Beijing, China) 2024-0001. The mice were raised in the experimental animal center of Beijing University of traditional Chinese medicine, and all animal experiments were approved by the Experimental Animal Ethics Committee of Beijing University of Chinese Medicine (No: BUCM-2025022502-1066). At the end of experiment, the mice were euthanized by cervical dislocation. During the operation, it was ensured that the movements were quick enough to minimize animal suffering to the greatest extent possible.

### 2.2. Processes for CUR@TA-Fe(III) NPs

Curcumin was dissolved in dimethyl sulfoxide to a concentration of 80 mg/mL. A total of 50 μL of the above solution was added to 2.4 mL of 0.1% PVP solution, and the mixture was stirred via a magnetic stirrer at 800 rpm for 20 min. Then, 100 μL of TA (46.2 mg/mL) and 60 μL of anhydrous FeCl_3_ (44 mg/mL) solution were added sequentially, and stirring was continued for 2 h. Then, the reaction mixture was centrifuged at 12,000 rpm for 15 min, the supernatant was discarded, and the reaction mixture was washed with deionized water three times to obtain CUR@ TA-Fe(III) NPs.

### 2.3. Drug Encapsulation Rate and Drug Loading Capacity

The amount of Curcumin in the nanoparticles was quantified by a high-performance liquid chromatography (HPLC) instrument (UltiMate U3000 high-performance liquid chromatography instrument (Thermo Fisher, Waltham, MA, USA)) equipped with an Agilent ZORBAX SB-C18 column (4.6 mm × 250 mm, 5 μm, Santa Clara, CA, USA). The detection wavelength was established at 430 nm. The mobile phase consisted of acetonitrile and water with 4% acetic acid in a ratio of 48:52 with a flow rate of 1 mL/min, and the encapsulation efficiency (EE) and LE of Curcumin in the nanoparticles were calculated according to the formulas given in Equations (1) and (2) [26,27,28].

EE (%) = (mass of drug in nanoparticles/mass of drug fed) × 100% (1)

LE (%) = (mass of drug in nanoparticles/total mass of nanoparticles) × 100% (2)

### 2.4. Characterization of CUR@TA-Fe(III) NPs

The particle size distribution and zeta potential of the CUR@TA-Fe(III) NPs, as well as the particle size of the nanoparticles over a period of seven days in the dark at room temperature, were determined via a Zeta Sizer Nano-ZS90 (Malvern Panalytical Instrument, Malvern, UK); the particle morphology and surface elemental distribution of the nanoparticles were observed via a field emission transmission electron microscope (Thermo Fisher-Talos F200S, Waltham, MA, USA) to observe the morphology of the particles and the distribution of the elements on the surface; and the nanoparticles were analyzed using a K-ALPHA X-ray photoelectron spectroscopy (XPS) instrument from US-Thermo Scientific (Waltham, MA, USA). The full-wavelength scans of CUR@TA-Fe(III) NPs, CUR NPs, and TA-Fe(III) in the range of 250–850 nm were measured via a dual-beam UV-visible spectrophotometer (TU-1901, Beijing Pudian General Instrument Co., Ltd., Beijing, China); the lyophilized powders were obtained via a lyophilizer (FD-2A, Beijing Bomikang Experimental Instrument Co., Ltd., Beijing, China) and analyzed via a Fourier exchange infrared spectrometer (TY2020009744, Jintuo Instrument Technology Co., Ltd., Tianjin, China) in the range of 400–4000 cm^−1^ for functional group analysis.

### 2.5. Photothermal Properties of CUR@TA-Fe(III) NPs

To investigate the photothermal effect of CUR@TA-Fe(III) NPs, samples with varying Curcumin concentrations were irradiated with an 808 nm laser at a power density of 2.0 W/cm^2^. Additionally, the samples, at a concentration of 50 μg/mL, were tested at different power densities (1.0, 1.5, and 2.0 W/cm^2^), with a spot diameter of 1 cm.

To assess the photothermal stability of CUR@TA-Fe(III) NPs, the samples, at a concentration of 50 μg/mL, were irradiated with an 808 nm laser at 2.0 W/cm^2^. Following irradiation, the laser was turned off to allow the samples to cool back to their initial temperature. This operational cycle was repeated three times. The temperature of each sample was recorded via an IR camera (Version Ax5, FLIR Systems, Inc., Wilsonville, OR, USA) and analyzed via FLIR Tools 5.13 versiom software.

### 2.6. Cellular Uptake

4T1 cells were initially seeded in confocal dishes and allowed to incubate overnight in an incubator. After the cells adhered to the wall, they were treated with free Coumarin 6 or Coumarin 6@TA-Fe(III) NPs. The intracellular fluorescence was subsequently captured and recorded via confocal laser scanning microscopy (CLSM) (Leica Microsystems, Wetzlar, Germany).

### 2.7. In Vitro Cytotoxicity

#### 2.7.1. Cytotoxicity Assay

The cytotoxicity of the different preparations was examined using a CCK-8 assay. 4T1 cells were seeded in 96-well plates and allowed to incubate for 24 h. Then, the cells were treated with different samples containing Curcumin at concentrations of 0, 5, 10, 20, 30, and 40 μg/mL for another 24 h. The CUR@TA-Fe(III) + laser group was irradiated (808 nm, 2 W/cm^2^) for 5 min at the 4th hour. Following treatment, the medium was replaced with 100 μL of CCK-8 solution (10%) and incubated for 0.5 h. Then, the absorbance of the solution was measured with a plate reader at a wavelength of 450 nm.

#### 2.7.2. Live/Dead Cell Assay

A live/dead cell staining kit was used to assess cellular growth following treatment with different formulations: Fe(III), TA, free Curcumin, TA-Fe(III)+/−laser, and CUR@TA-Fe(III) NP+/−laser. The cells were washed with PBS and stained with 300 μL of calcein AM/PI solution for 30 min. The live/dead state of the cells was observed via CLSM.

### 2.8. In Vitro Ferroptosis Mechanism and Photothermal–Ferroptosis Therapy

#### 2.8.1. Intracellular Fe(II) Detection

A FerroOrange probe was used to examine the level of Fe(II) in 4T1 cells. 4T1 cells were incubated with different formulations as described in Section 2.7.2. The cells were stained with a FerroOrange probe for 30 min. The Fe(II) levels of each group were determined via CLSM.

#### 2.8.2. Intracellular ROS Detection

A DCFH-DA probe was used to examine the level of ROS production in 4T1 cells. 4T1 cells were incubated with different formulations as in Section 2.7.2, and then stained with a DCFH-DA probe for 20 min. The ROS levels in each group were determined via CLSM.

#### 2.8.3. Intracellular LPO Detection

To evaluate the extent of ferroptosis, C11-Bodipy 581/591 was selected as the LPO probe. After incubation with different formulations for 24 h, the cells were stained with C11-BODIPY 581/591 for 30 min and then observed by fluorescence microscopy, and Image J 1.53 t was used to analyze the images quantitatively.

#### 2.8.4. Intracellular Mitochondrial Membrane Potential Detection

To evaluate the extent of ferroptosis, JC-1 was used to detect changes in the mitochondrial membrane potential (MMP). After incubation with different formulations for 24 h, the cells were treated with JC-1 solution for 30 min and then observed by fluorescence microscopy, and Image J 1.53 t was used to analyze the images quantitatively.

#### 2.8.5. GSH Level Detection

The GSH kit was used to test the GSH levels in 4T1 cells. The amount of GSH was quantified via an enzyme marker at 412 nm, and the relative GSH level of each group was subsequently obtained by dividing each group’s GSH content by the amount of GSH in the control group.

### 2.9. In Vivo Antitumor Effects

#### 2.9.1. In Vivo Photothermal Efficacy

4T1 cells (100 μL, 1 × 10^7^/mL) were subcutaneously injected into the right side of the backs of BALB/c mice to establish tumor models. The mice were chosen at random and given an intratumor injection of either saline or CUR@Fe-TA(III) once the tumor volume reached approximately 300 mm^3^. The mouse tumor area was then exposed to an 808 nm laser at 2 W/cm^2^ for 5 min. An IR camera was used to simultaneously record the temperature of the tumor area.

#### 2.9.2. Antitumor Effect

When the tumor volume reached 100 mm^3^, mice were randomly assigned to four groups (n = 6). The mice subsequently received 100 µL of saline, free Curcumin, Cur@TA-Fe(III), or Cur@TA-Fe(III) + laser (5 mg·kg^−1^ Curcumin) intratumorally at intervals of three days and twice in total. The body weight and tumor volume were measured every two days, and the formula V = (L × W^2^)/2 (V = tumor volume, L = tumor length, W = tumor width) was used to compute the tumor volume. On the final day, the tumor growth inhibition (TGI) rate was computed on the basis of tumor volume. All of the mice were killed on the fifteenth day, and the tumors were removed so that they could be weighed and photographed. Then, the tumors were fixed in 4% paraformaldehyde and stained with H&E and TUNEL.

## 3. Results and Discussion

### 3.1. Processes for CUR@TA-Fe(III) NPs

**Figure 1:**
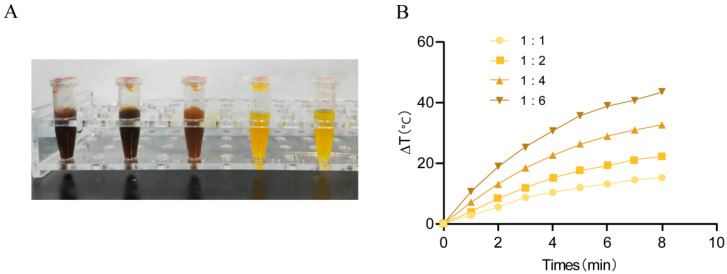
Preparation of CUR@TA-Fe(III) NPs. (**A**) Dispersions of CUR@TA-Fe(III) nanoparticles prepared with 0.5% concentrations of different stabilizers (from left to right: PVA, PVP, HPMC, F68, and F127). (**B**) Increase in photothermal temperature of CUR@TA-Fe(III) NPs with different molar ratios of TA to FeCl_3_ with 0.1% PVP.

Curcumin solution was added to aqueous solutions containing 0.5% PVA, 0.5% PVP, 0.5% HPMC, 0.5% F68, and 0.5% F127 as stabilizers. Following the addition of Curcumin, when F68 and F127 were used as stabilizers, the TA-Fe(III) coordination complex could not be well coated on the particle surface (Figure 1A). The particle size, drug loading capacity, and encapsulation efficiency of the nanoparticles prepared with 0.5% PVA, 0.5% PVP, and 0.5% HPMC were subsequently investigated. HPMC is a type of polymer derived from cellulose, PVA is a polymer consisting of vinyl alcohol units, and PVP is a polymer composed of N-vinyl-2-pyrrolidone units. In particular, the carbonyl groups in PVP’s structure could serve as hydrogen bond acceptors and interact with polyphenols through dipole–dipole, charge transfer or hydrophobic interaction [29]. Wegiel L.A. [30] compared the strength of hydrogen bonds between HPMC-CUR and PVP-CUR in curcumin suspensions via Fourier infrared spectroscopy. It was found that the hydrogen bonding between cellulose polymers and Curcumin was very weak, whereas PVP formed strong hydrogen bonding with curcumin, which means that it could adhere to the curcumin nanoparticle surface more firmly, thus providing a better stabilizing effect. Our study showed similar result. When using 0.5% HPMC to prepare the curcumin NPs, significant sedimentation was observed after two hours of storage. In contrast, curcumin NPs prepared with 0.5% PVA exhibited a smaller particle size (188.8 nm) and good dispersing stability. For the group utilizing 0.5% PVP as a stabilizer, curcumin NPs with the smallest particle size (124.7 nm) were obtained, along with the highest EE%, which could be attributed to the strong hydrogen bonding between PVP and Curcumin. The detailed experimental data were presented in Table 1.

The concentration of the stabilizer was also optimized. At PVA concentrations of 0.1% and 0.5%, the particle size was identically small. However, when the PVP concentration increased from 0.1% to 1%, the LE% showed a decreasing trend. An amount of 0.1% PVP showed the highest LE%. We did not further decrease the PVP concentration lower than 0.1%, since aggregation may occur due to insufficient steric hindrance [31,32]. Therefore, 0.1% was determined to be the optimal PVP concentration. The detailed experimental data were presented in Table 2.

To meet the requirements of subsequent cellular experiments, the nanosuspension was diluted to a concentration of 50 μg/mL [28]. The results for the photothermal effect showed that the nanosuspension achieved a temperature increase of 15 °C within 8 min under 808 nm laser irradiation. To optimize the heating efficiency, the molar ratio of TA to FeCl_3_ was determined. As depicted in Figure 1B, increasing the molar ratio of TA:FeCl_3_ from 1:1 to 1:6 increased the heating capacity. It is speculated that more Fe(III) combined with TA, thereby promoting the deposition of TA-Fe(III) complexes on the surface of the nanoparticles. Dong [33] constructed a TA-Fe(III) coating on the surface of MIL-101(Fe(III)). Their research revealed that the molar ratio of tannic acid to Fe(III) significantly influenced particle size. As this ratio increased, the particle sizes of MIL-101(Fe(III)) @TA, MIL-101(Fe(III)).)@TA-Fe(III)). (1:1), MIL-101(Fe(III)).@TA-Fe(III) (1:2), and MIL-101(Fe(III))@TA-Fe(III) (1:3) progressively increased. This indicates that the introduction of iron ions and an increased proportion thereof facilitate the formation of a TA-Fe(III) composite layer on the surface of nanoparticles. However, the LE decreased to 15.50 ± 0.67% (Figure 1B). This occurred because more TA-Fe(III) was deposited on the surface at this molar ratio, and the increased weight of TA-Fe(III) consequently led to a reduction in the Curcumin LE% according to the calculation formula. When the concentration of the Curcumin solution was doubled, the particle size decreased from 351.8 ± 2.8 nm to 169.9 ± 1.2 nm, and the LE increased to 27.99 ± 0.92%. This could be because more Curcumin nanoparticles were formed and coated with the same amount of TA-Fe(III) complexes. Moreover, the maximum temperature of the nanoparticles under the 808 nm laser slightly decreased.

When free Curcumin dissolved in DMSO was dropped into water, the hydrophobic curcumin molecules underwent self-assembly into CUR NPs through hydrophobic interactions and hydrogen bonds during the solvent exchange process. Afterwards, the added TA formed a coordinate complex with Fe(III) through its inherent pyrogallol and catechol groups. Then the TA-Fe(III) complex was deposited on the curcumin nanoparticle surface and formed a coating layer via π-π stacking, hydrogen bonding, and hydrophobic interaction to yield TA-Fe(III)-coated Curcumin nanoparticles, referred as CUR@TA-Fe(III) NPs. The CUR@TA-Fe(III) NPs can gradually release curcumin within tumor cells to exert chemotherapeutic effects. The TA-Fe(III) coating layer can perform PTT under NIR light irradiation. Additionally, TA-Fe(III) can bring Fe(III) to tumor microenvironments with a high H_2_O_2_ level. Fe(III) can be reduced to Fe(II) by TA, and then Fe(II) can catalyze the Fenton reaction and generate ROS, potentially inducing ferroptosis. The TA-Fe(III) network can continuously provide Fe(III) and Fe(II). In total, CUR@TA-Fe(III) NPs are expected to achieve better therapeutic efficacy against tumors by integrating CT, PTT, and ferroptosis. It is also worth mentioning that the photothermal performance of TA-Fe(III) was closely correlated to its concentration; therefore, adequate accumulation of TA-Fe(III) within tumors is necessary to guarantee effective thermal ablation of tumors.

### 3.2. Characterization of CUR@Fe-TA(III) NPs

**Figure 2:**
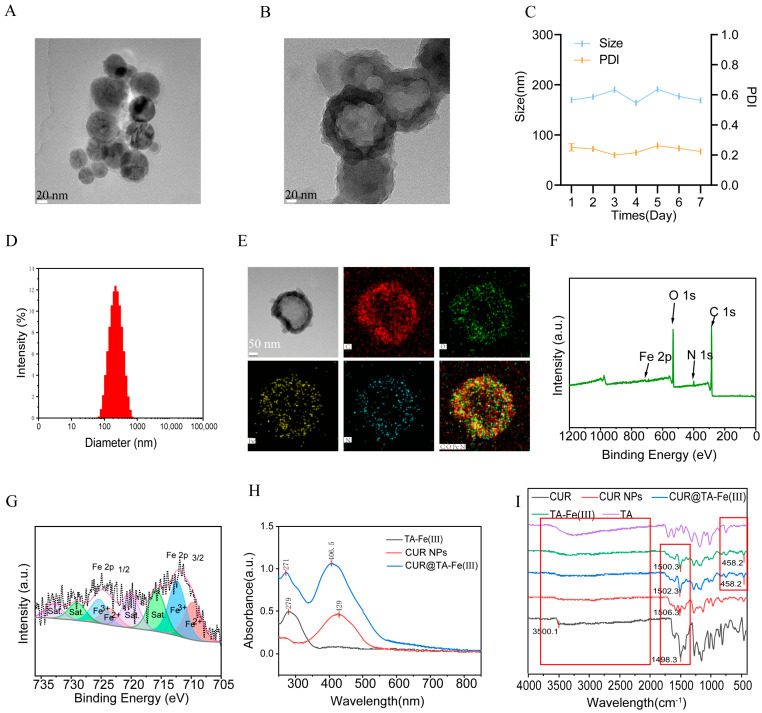
Characterization of CUR@TA-Fe(III) NPs. (**A**) TEM image of CUR NPs (scale bar: 20 nm). (**B**) TEM image of CUR@TA-Fe(III) NPs (scale bar: 20 nm). (**C**) Seven-day stability test results of CUR@TA-Fe(III) NPs (RT, dark). (**D**) Size distribution of CUR@TA-Fe(III) NPs. (**E**) TEM image and EDS results of CUR@TA-Fe(III) NPs (scale bar: 50 nm). (**F**) Full XPS spectrum of CUR@TA-Fe(III) NPs. (**G**) High-resolution Fe 2p spectra of CUR@TA-Fe(III) NPs. (**H**) UV-Vis spectrogram. (**I**) FTIR spectrogram.

Figure 2A showed that the CUR NPs were spherical particles. As shown in Figure 2B, TA-Fe(III) was coated around the CUR NPs. Generally, a higher zeta potential is linked to better particle stability, as it promotes charge repulsion between particles, preventing aggregation and dispersion [34]. In the dispersion stability test, the particle size of the CUR@TA-Fe(III) NPs indeed changed little, and the PDI was always 0.2 over 7 days (Figure 2C). These results suggest that the stability of Curcumin self-assembled at the nanoscale could be improved after TA-Fe(III) coating. TA-Fe(III) created steric hindrance to prevent aggregation. Also, the zeta potential of curcumin nanoparticles dropped significantly from −17.1 mV to −42.5 mV after TA-Fe(III) coating, which was due to the negatively charged catechol groups in TA. This could enhance the electrostatic repulsion between nanoparticles to avoid aggregation.

Next, the physical properties of the CUR@TA-Fe(III) NPs were examined. The diameter of the CUR@TA-Fe(III) NPs was 169.9 ± 1.2 nm (Figure 2D). Figure 2E shows the energy-dispersive X-ray spectroscopy (EDS) results, which revealed that C, O, N, and Fe were distributed on the surface. As shown in Figure 2F and 2G, the valence analysis of Fe revealed that some of the Fe(III) was converted to Fe(II).

UV-Vis spectroscopy revealed that the absorption peak of CUR@TA-Fe(III) was characteristic of both Curcumin nanoparticles and TA-Fe(III), indicating that the TA-Fe(III) coating did not significantly alter the absorption of Curcumin (Figure 2H). The spectrum of CUR@TA-Fe(III) also showed two clear blueshifts in the peak assigned to TA (from 279 to 271 nm) and another in the one assigned to Curcumin (from 429 nm to 406.5 nm). This phenomenon was possibly due to the π-π stacking and H-aggregation formed between the aromatic structure in TA and Curcumin [35,36].

The FTIR spectrum revealed that the broad stretching vibration peak (3600–3000 cm^−1^) of phenolic hydroxyl groups in TA showed a weakened signal in comparison with TA-Fe(III). The presence of the Fe-O bending vibration peak at 458.2 cm^−1^ confirmed the coordination interaction between Fe(III) and TA [37] (Figure 2I). Compared to crude curcumin, the phenolic hydroxyl stretching peak that originally appeared at 3500.1 cm^−1^ became weakened and transformed into a broad peak (3600–3000 cm^−1^) for the CUR NPs, possibly due to the hydrogen bonds that formed between curcumin and PVP during self-assembly. CUR@TA-Fe(III) retained the characteristic peaks of both CUR NPs and TA-Fe(III), with the benzene ring vibration peak (1502.3 cm^−1^) showing higher intensity than in CUR NPs (1506.3 cm^−1^) and TA-Fe(III) (1500.3 cm^−1^).

**Figure 3:**
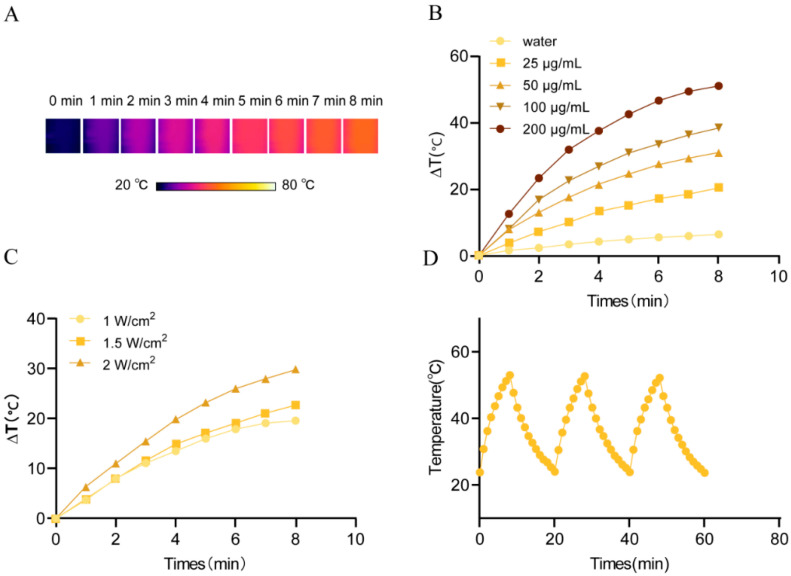
Photothermal effects of CUR@TA-Fe(III) NPs. (**A**) Thermal images of CUR@TA-Fe(III) NPs (the concentration of CUR was 50 μg/mL) under laser irradiation (808 nm, 2 W/cm^2^). (**B**) Increase in temperature of CUR@TA-Fe(III) NPs at different CUR concentrations under laser irradiation (808 nm, 2 W/cm^2^). (**C**) Increase in temperature of CUR@TA-Fe(III) NPs (the concentration of CUR was 50 μg/mL) at different power densities. (**D**) Photostability of CUR@TA-Fe(III) NPs during three cycles of laser irradiation.

The photothermal performance of the CUR@TA-Fe(III) nanoparticles could be tuned by adjusting the nanoparticle concentration and laser power density, resulting in excellent stability and reproducibility (Figure 3A–D).

### 3.3. Cellular Uptake

**Figure 4:**
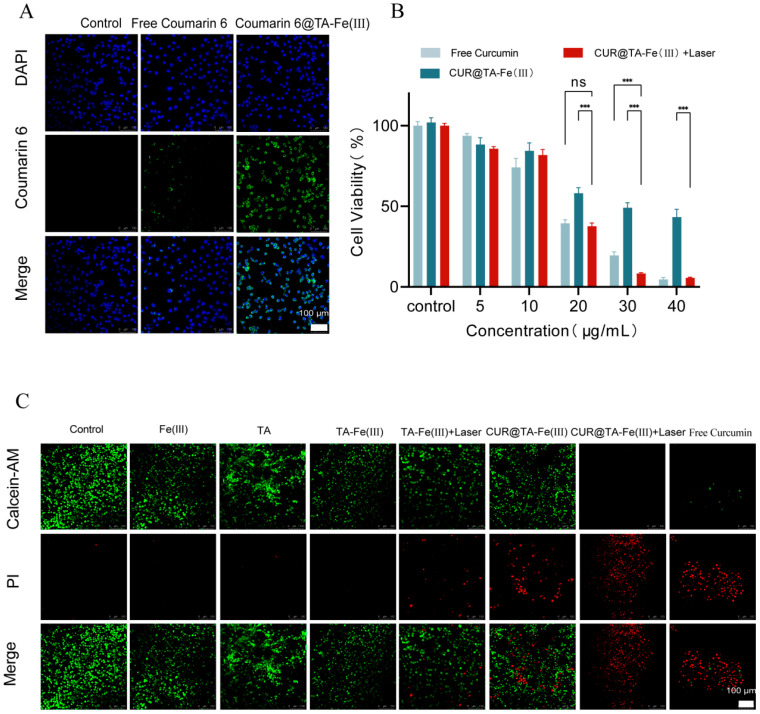
In vitro evaluation of treatment efficacy. (**A**) Fluorescence images of 4T1 cells showing the uptake of CUR@TA-Fe(III) (scale bar: 100 μm). (**B**) Viability of 4T1 cells in different formulation groups (n = 5) (statistical analysis: ns indicates *p* > 0.05 and *** indicates *p* < 0.001). (**C**) Confocal images of 4T1 cells stained with calcein-AM/PI for live/dead staining. (scale bar: 100 μm).

To investigate cellular internalization, CUR@TA-Fe(III) NPs were labeled with fluorescent coumarin 6 (C6). As shown in Figure 4A, after 4 h of treatment, green fluorescence was observed in the free coumarin 6 and coumarin 6@TA-Fe(III) NP groups compared with the control group. Compared with the free Coumarin 6 group, the Coumarin 6@TA-Fe(III) NP group presented a stronger green fluorescence signal, indicating that TA-Fe(III) significantly enhanced drug internalization.

### 3.4. In Vitro Cytotoxicity

CCK-8 assays were conducted to assess the cytotoxicity of CUR@TA-Fe(III) on 4T1 cells. As illustrated in Figure 4B, free Curcumin exhibited dose-dependent cytotoxicity within the range of 0–40 μg/mL. At the concentration of 20 μg/mL, there is no significant difference in cell viability between the free-Curcumin group and the CUR@TA-Fe(III) + laser group. Firstly, the cell viability of the CUR@TA-Fe(III) group was much higher than that of the free-curcumin group, which may be due to the slow release of Curcumin resulting from the hindrance of the TA-Fe(III) coating. Meanwhile, after laser irradiation, the cell viability CUR@TA-Fe(III) + Laser group markedly decreased and became similar to that of the free-Curcumin group. When we further increased the concentration to 30 μg/mL, the cell viability of the CUR@TA-Fe(III) + laser group (8.4%) was significantly lower than that of the free-Curcumin group (19.5%), indicating that the TA-Fe(III) mediated PTT, and Curcumin synergistically enhanced the cytotoxic effects against the 4T1 cells.

Calcein-AM/PI double staining further confirmed the cytotoxic effects (Figure 4C). Strong green fluorescence and minimal red fluorescence were observed in the control, Fe(III), TA, and TA-Fe(III) groups, indicating that the TA-Fe complex has no toxicity. The TA-Fe(III) + laser group showed moderate red fluorescence, which was attributed to the hyperthermia of TA-Fe(III). The CUR@TA-Fe(III) group also presented moderate red fluorescence, which was lower than that of the free-Curcumin group and was consistent with the results of the CCK-8 assay. In contrast, CUR@TA-Fe(III) + laser resulted in intense red fluorescence, indicating that the synergy of the photothermal and chemotherapeutic effects could potently kill 4T1 cells.

### 3.5. In Vitro Ferroptosis Mechanism and Photothermally Strengthened Ferroptosis

**Figure 5:**
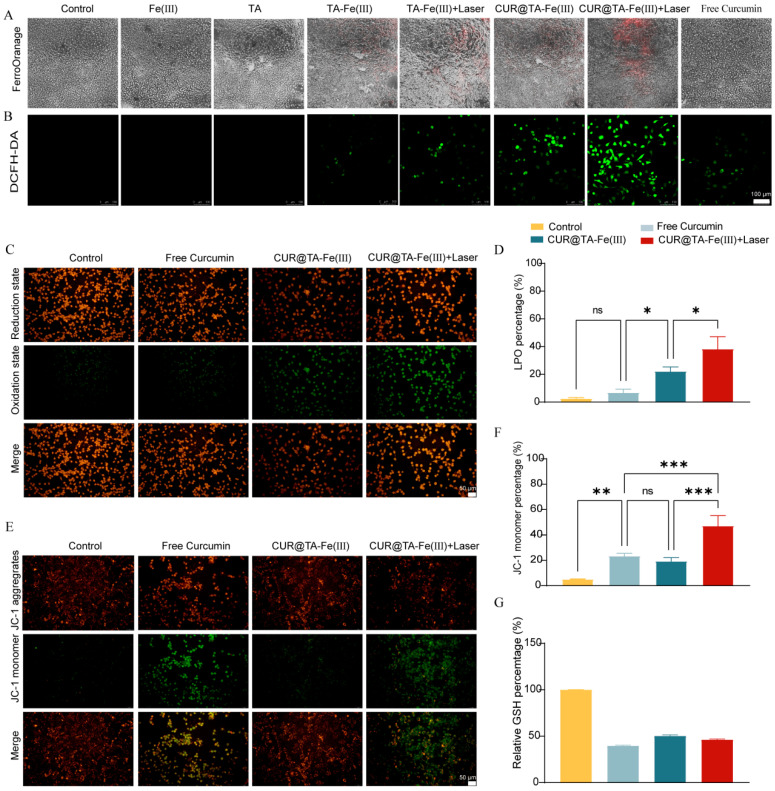
In vitro ferroptosis mechanism and photothermal strengthening of ferroptosis. (**A**,**B**) Confocal images of 4T1 cells stained with the FerroOrange probe and DCFH-DA probe (scale bar: 100 μm). (**C**) Fluorescence images of 4T1 cells stained with BODIPY 581/591 C11 probe after treatment with different formulations (scale bar: 50 μm). (**D**) Semiquantitative analysis of fluorescence images of BODIPY 581/591 C11 probe-stained samples via ImageJ software. (**E**) Fluorescence images of 4T1 cells stained with the JC-1 probe after treatment with different formulations (scale bar: 50 μm). (**F**) Semiquantitative analysis of fluorescence images stained with the JC-1 probe via ImageJ software. (**G**) Relative content of intracellular glutathione (GSH) in 4T1 cells after treatment with different formulations (statistical analysis: ns indicates *p* > 0.05, * indicates *p* < 0.05, ** indicates *p* < 0.01, and *** indicates *p* < 0.001).

Free Fe(II) can be detected by a FerroOrange probe, which emits red fluorescence (Figure 5A). The control group, Fe(III) group, TA group, and free-Curcumin group exhibited no red fluorescence, indicating that the same concentrations of Fe(III), TA, and Curcumin as those in the NPs had no obvious effect on the intracellular Fe(II) levels. The TA-Fe(III) group displayed weak red fluorescence, suggesting that a small amount of Fe(III) was reduced to Fe(II) by TA. In contrast, the TA-Fe(III) + laser group showed obvious red fluorescence, indicating that PTT promoted the conversion of Fe(III) to Fe(II). When compared with the TA-Fe(III) group, the CUR@TA-Fe(III) group demonstrated similar red fluorescence intensities. However, the CUR@TA-Fe(III) + laser group exhibited a much stronger intensity and larger area of red fluorescence than the TA-Fe(III) + laser group, implying elevated intracellular levels of Fe(II).

As illustrated in Figure 5B, Fe(II) catalyzes and participates in the Fenton reaction: Fe(II) + H_2_O_2_→ Fe(III) +·OH + OH^−^. This ultimately leads to a ROS storm [38]. Following DCFH-DA staining, intracellular ROS are indicated by green fluorescence. The control group, the Fe(III) group, and the TA group exhibited almost no green fluorescence. Weak green fluorescence was observed in the TA-Fe(III) group as a consequence of the Fenton reaction. In contrast, half of the cell area in the TA-Fe(III) + laser group displayed green fluorescence, suggesting that PTT enhanced ferroptosis to generate an increased level of ROS [39]. The free-Curcumin group also presented obvious green fluorescence, indicating that Curcumin induced ROS generation in 4T1 tumor cells, which was consistent with the findings of several previous studies [40]. Compared with the TA-Fe(III) and free-Curcumin groups, the CUR@TA-Fe(III) group exhibited stronger green fluorescence. Compared with the other groups, the CUR@TA-Fe(III) + laser group showed the most intense green fluorescence, indicating that most of the ROS were generated.

These results indicate that PTT can strengthen the ferroptosis mediated by TA-Fe(III) through the elevation of intracellular Fe(II) levels, thereby facilitating efficient production of ROS. Furthermore, curcumin was found to induce ROS generation within cells, despite having no effect on the intracellular Fe(II) level. For the CUR@TA-Fe(III) NPs, the dual mechanisms of Curcumin and TA-Fe(III) resulted in a substantial increase in Fe(II) and ROS production, significantly elevating oxidative stress levels.

In the ROS and Fe(II) detection experiments, neither the Fe(III) nor the TA group influenced the intracellular Fe(II) levels or induced ROS production. Consequently, these groups were excluded from subsequent experiments. Three experimental groups were established: free Curcumin, CUR@TA-Fe(III), and CUR@TA-Fe(III) + laser.

An increase in the ROS level could lead to lipid peroxidation and the subsequent production of substantial amounts of LPO, causing a shift in the fluorescence of the Bodipy 581/591 C11 probe from red to green. As illustrated in Figure 5C and 5D, the ratio of green fluorescence to total fluorescence (green + red) in the free-Curcumin, CUR@TA-Fe(III), and CUR@TA-Fe(III) + laser groups was higher than the ratio in the control group, indicating an elevated presence of LPO within cells. Notably, when compared with the free-Curcumin group, the CUR@TA-Fe(III) group exhibited greater green fluorescence intensity, suggesting more LPO in cells. Furthermore, upon application of 808 nm laser irradiation, the CUR@TA-Fe(III) group displayed the most obvious green fluorescence, indicating that a significant amount of LPO was produced. This observation further corroborates that PTT can augment ferroptosis [41].

When mitochondria function normally, the difference in the membrane potential remains constant, and JC-1 aggregates emit red fluorescence. Conversely, a decrease in membrane potential leads to the presence of JC-1 monomers that emit green fluorescence. In this study, the ratio of green fluorescence to total fluorescence (green + red) was used as an indicator of MMP status (Figure 5E,F). Compared to the control group, the ratio in the free-Curcumin group increased, suggesting that mitochondrial damage occurred following treatment. The CUR@TA-Fe(III) group exhibited lower levels of green fluorescence than the free-Curcumin group; this may be attributed to a slow-release effect. Furthermore, there was no significant difference between the CUR@TA-Fe(III) + laser group and the free-Curcumin group, consistent with cell viability measurements. As shown in Figure 5G, GSH levels were significantly reduced in the three experimental groups compared to those observed in the control group. These findings suggest that CUR@TA-Fe(III) NPs could deplete intracellular GSH.

In conclusion, CUR@TA-Fe(III) NPs with NIR light irradiation effectively induced ferroptosis in 4T1 cells by elevating the intracellular Fe(II), ROS, and LPO levels. These NPs provide a new strategy for achieving synergistic effects in tumor treatment.

### 3.6. Antitumor Activity In Vivo

#### 3.6.1. In Vivo Photothermal Warming Effect

**Figure 6:**
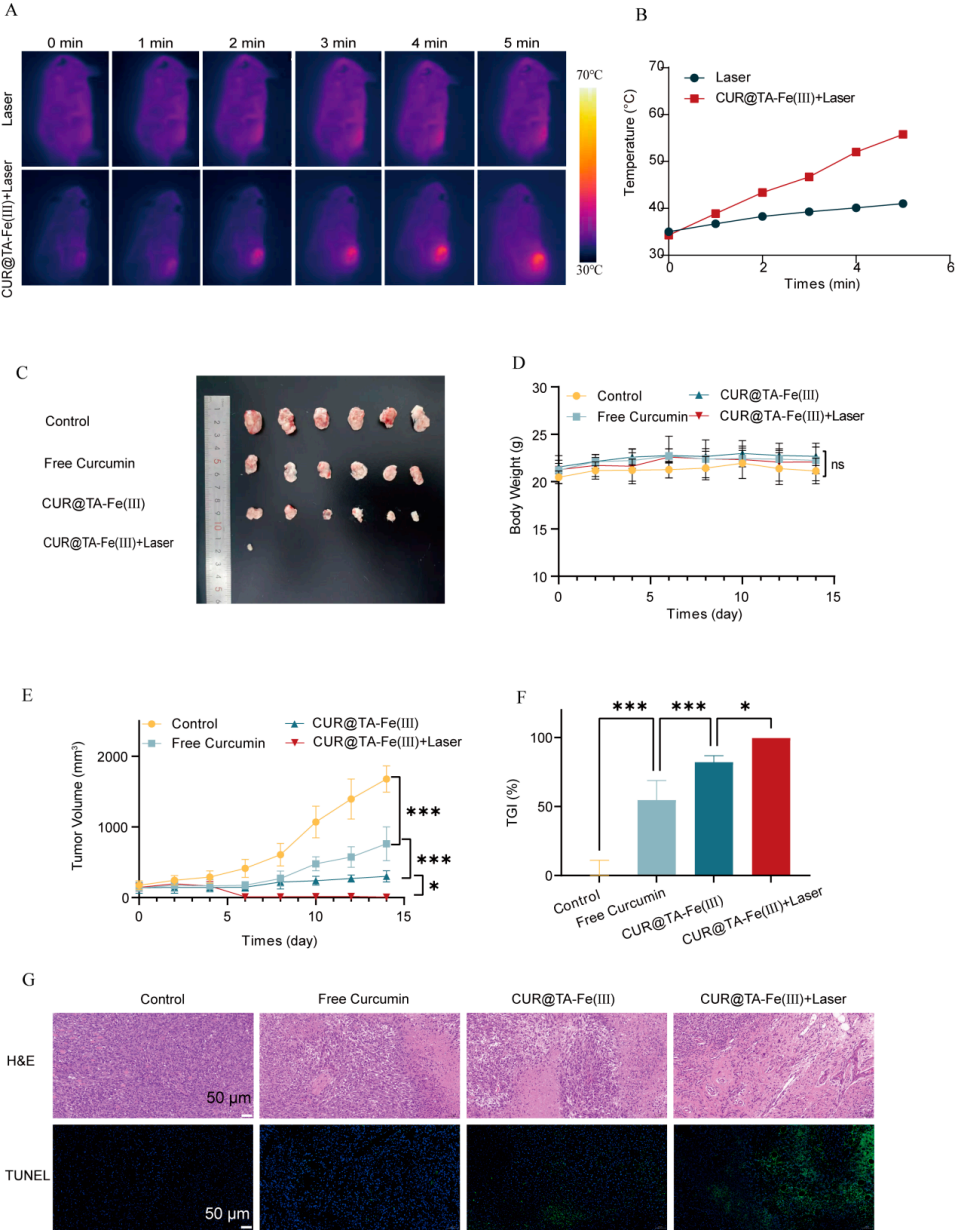
Investigation of the in vivo antitumor efficacy of CUR@TA-Fe(III) nanoparticles (NPs). (**A**) Thermal imaging images of the tumors. (**B**) Curves of local tumor temperature elevation. (**C**) Photographs of tumors from different treatment groups 14 days after treatment. (**D**) Curves of body weight changes in experimental animals (statistical analysis: ns indicates *p* > 0.05, * indicates *p* < 0.05, and *** indicates *p* < 0.001). (**E**) Curves of tumor volume changes. (**F**) TGI of different treatment groups 14 days after treatment. (**G**) Staining results of tumor tissue sections from different treatment groups 14 days after treatment, including HE staining and TUNEL staining.

As shown in Figure 6A,B, laser irradiation induced a temperature increase from 35.0 to 41.0 °C in the control group’s tumor area. In contrast, the temperature of the CUR@Fe-TA(III)-treated mice increased from 34.3 to 55.8 °C. This temperature increase exceeds the threshold required for tumor cell ablation, confirming the in vivo photothermal efficacy of CUR@Fe-TA(III) [42].

#### 3.6.2. In Vivo Antitumor Effects

During the treatment period, no significant changes in body weight were observed within or between groups, indicating that the nanoparticles exhibited no systemic toxicity (Figure 6D). In terms of tumor volume (Figure 6C,E), the free-Curcumin, CUR@TA-Fe(III), and CUR@TA-Fe(III) + laser groups presented greater inhibition of tumor growth than the control group. Following the second administration, the CUR@TA-Fe(III) group showed a smaller increase in tumor volume than the free-Curcumin group, likely due to increased tumor penetration attributed to the increased tumor penetration of nanoscale particles. In contrast, the laser group achieved tumor ablation after the first treatment and nearly complete tumor elimination following the second treatment, thereby confirming its superior efficacy. The TGI followed the same trend (Figure 6F).

Ex vivo tumor tissues were analyzed via H&E and TUNEL staining (Figure 6G). The H&E staining results indicated that the tissue architecture of each group exhibited varying degrees of damage: the control group maintained a compact tissue structure, while the free-Curcumin group displayed moderate damage, the CUR@TA-Fe(III) group showed more pronounced tissue destruction, and the CUR@TA-Fe(III) + laser group exhibited extensive and evident tissue damage, characterized by a significantly looser tissue structure. TUNEL staining was employed to assess the percentage of apoptotic cells, and no signal was detected in the control group. Weak fluorescence was observed in the free-Curcumin group and the CUR@TA-Fe(III) group, whereas intense fluorescence signals were detected in the CUR@TA-Fe(III) + laser group, confirming enhanced induction of apoptosis through combination therapy.

Upon 808 nm laser irradiation, CUR@TA-Fe(III)NPs induced localized tumor hyperthermia, selectively ablating tumor tissues while sparing adjacent normal tissues. The combination of PTT and CT demonstrated superior efficacy in tumor suppression compared to CT alone.

## 4. Conclusions

In this study, we prepared CUR@TA-Fe(III) NPs, which exhibited high drug LE% and remarkable photothermal properties and stability. In addition to the inhibitory effect of Curcumin on the growth of cancer cells, the TA-Fe(III) coating efficiently converted NIR light energy into heat, leading to direct thermal ablation of 4T1 cells. Additionally, the TA-Fe(III) network induced ferroptosis by increasing the intracellular Fe(II) content and generating massive amounts of ROS through the Fenton reaction. PTT was found to be able to strengthen this effect. Curcumin also generated ROS and indirectly promoted ferroptosis. Therefore, CUR@TA-Fe(III) NPs integrated chemotherapy, photothermal therapy, and ferroptosis. This multimodal treatment could overcome the limitations of traditional single-treatment methods and provide a more effective strategy to improve the therapeutic outcomes of TNBC patients.

## Figures and Tables

**Table 1:** Effects of different stabilizers on CUR@TA-Fe(III) nanoparticles (n = 3).

Stabilizer	Particle Size (nm)	PDI	EE (%)	LE (%)
0.5% PVA	188.8 ± 0.6	0.157 ± 0.009	41.33 ± 2.80	13.53 ± 0.63
0.5% PVP	124.7 ± 0.8	0.178 ± 0.007	60.24 ± 1.04	9.49 ± 0.17
0.5% HPMC	343.7 ± 3.6	0.219 ± 0.008	43.83 ± 7.67	10.09 ± 1.06

**Table 2:** Effect of stabilizer concentration on CUR@TA-Fe(III) nanoparticles (n = 3).

Stabilizer	Particle Size (nm)	PDI	EE (%)	LE (%)
0.1% PVP	124.2 ± 0.7	0.227 ± 0.039	52.09 ± 0.21	21.97 ± 0.36
0.5% PVP	124.7 ± 0.8	0.178 ± 0.007	60.24 ± 1.04	9.49 ± 0.17
1% PVP	203.8 ± 1.5	0.181 ± 0.009	46.70 ± 2.99	7.98 ± 0.22

## Data Availability

All data can be found either in this article. No artificial intelligence tools were used (neither to design this research nor to execute, analyze, or write it).
